# Lateral Decubitus Supraclavicular Brachial Plexus Block in Transverse Myelitis

**DOI:** 10.7759/cureus.47142

**Published:** 2023-10-16

**Authors:** Hemanthkumar Tamilchelvan, Shashank Paliwal, Upma Chugh, Anju Grewal

**Affiliations:** 1 Anesthesia, All India Institute of Medical Sciences, Bathinda, IND

**Keywords:** usg-guided nerve block, spastic paralysis, transverse myelitis, lateral decubitus position, supraclavicular brachial plexus block

## Abstract

Transverse myelitis is a rare inflammatory condition typically presenting with symptoms like muscle weakness, sensory issues, and problems affecting bowel and bladder function. In this study, we describe the successful anesthesia management of an adult patient with transverse myelitis exhibiting spastic paralysis and compromised cardiopulmonary reserves, whose preferred resting position was lateral decubitus. Targeted anesthesia was administered via a supraclavicular approach to the brachial plexus block for wrist deformity fixation surgery, mitigating the pulmonary complications associated with general anesthesia, achieving earlier recovery, and avoiding the use of opioids. This case underscores the significance of customizing the patient’s personalized positioning, while also highlighting the potential for effective regional anesthesia in atypical positions. We illustrate the successful use of supraclavicular brachial plexus block for left wrist deformity fixation and debridement surgery in the lateral decubitus, the most convenient position for the transverse myelitis patient with spastic paraplegia.

## Introduction

Transverse myelitis is a focal inflammatory disorder that commonly manifests with clinical presentations encompassing muscle weakness, sensory deficits, and complications related to bowel and bladder function [1]. This disorder occurs due to progressive loss of the fatty myelin sheath that surrounds the nerves in the affected spinal cord usually central, uniform, and symmetric. Spinothalamic tracts, pyramidal tracts, posterior columns, and anterior fasciculi are usually involved at one or more than one adjacent level. It affects both sexes with an incidence of between one and eight new cases per million per year with bimodal peak between the ages of 10-19 years and 30-39 years [2]. Autonomic dysreflexia can occur in transverse myelitis following general anesthesia giving rise to exaggerated hemodynamic variations and arrythmias [3]. Further administering general anesthesia in a patient with poor cardiopulmonary reserve manifests in hemodynamic instability and delayed recovery. The decision to perform regional anesthesia in these patients with pre-existing neurological disorders should be based on the risks of providing general anesthesia versus the benefits of regional anesthesia in each individual case [4]. Thus, anesthetic management in such patients also has a unique challenge of patient positioning to be considered. Supraclavicular brachial plexus block is a regional anesthetic technique for upper extremities surgery that blocks the brachial plexus from the distal trunks to the proximal blocks [5]. It is usually provided in the supine or semi-sitting position with head turned towards the opposite side and it provides pain relief to the distal two-thirds of the upper extremity [6].

## Case presentation

A 41-year-old female patient, who is a known case of transverse myelitis with spastic paraplegia for the past 13 years with a history of Pott's spine treated with the complete course of anti-tubercular therapy for 18 months, presented to the emergency room with an ulcer on the left deformed wrist with a discharging sinus. She was a known case of rheumatoid arthritis for the past 15 years with poor compliance with medications. Her past surgical history revealed that she had undergone a cesarean section under spinal anesthesia and a cholecystectomy under spinal anesthesia. Physical examination revealed that she could not stand, sit, or lie down in a supine position due to limitations from transverse myelitis. She had urinary and fecal incontinence. On examination, her heart rate was 110 beats per minute, blood pressure was 132/88 mmHg. Her airway examination revealed that the inter-incisor gap is 2 cm with restricted neck movements, decreased cervical spine mobility, class I upper lip bite test, modified Mallampati Class IV, thyromental distance 6.8 cm, and neck circumference 37 cm. She was concluded to have an anticipated difficult airway. She was conscious and oriented to time, place, and person. However, there was notable generalized spasticity, particularly in her lower limbs. Cranial nerves examination was normal. No cerebellar or meningeal irritation signs were detected. During the respiratory examination, bilateral basal crepitations more pronounced on the right side were observed upon auscultation. The erythrocyte sedimentation rate test and C-reactive protein test were raised, and other routine blood investigations were normal.

Magnetic resonance imaging with wall shear stress revealed a long-segment T2 hyperintense signal involving the spinal cord extending from C2 to D2 level, likely longitudinally extensive transverse myelitis with syrinx formation in its distal part (D7-D9 levels). The patient was planned for debridement and wrist deformity fixation. We decided to proceed with regional anesthesia with a supraclavicular brachial plexus block. Complete preparation for inducing general anesthesia, difficult airway cart, and drugs needed in case of autonomic hyperreflexia were kept ready.

**Figure 1:**
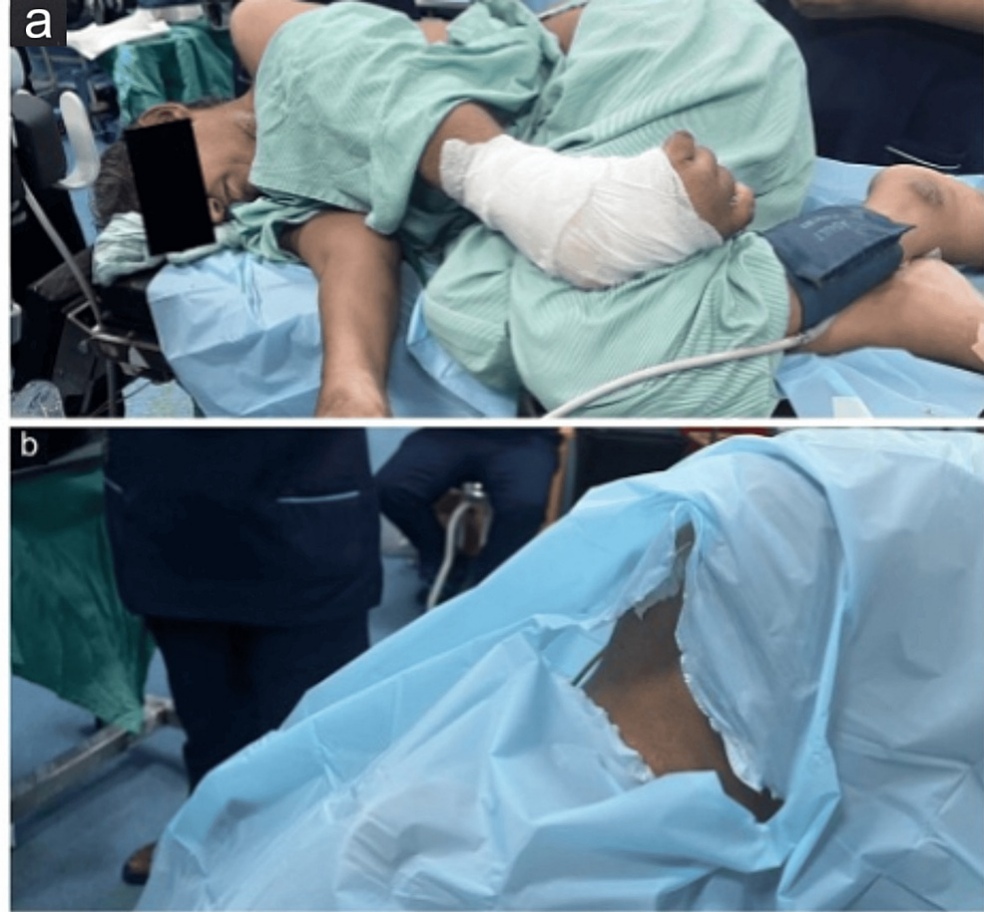
The patient is in the preferred resting (lateral decubitus) position (a). The exposed site for the supraclavicular block is in the lateral decubitus position (b).

The patient was kept nil per oral for 6 hours for light meals and 2 hours for clear liquids. Upon arrival to the operation room after consent for regional and general anesthesia, the patient was shifted to the operation table and made to lie in the lateral decubitus position due to the patient's inability to lie supine (Figures 1a, 1b). In the lateral decubitus position, a pillow was kept under the head, and a pillow was positioned in front of her thorax to place her comfortably. She was monitored according to the basic standards for anesthetic monitoring from the American Society of Anesthesiologists. A multiparameter monitor was used to monitor oxygen saturation, continuous electrocardiography, noninvasive blood pressure with a cuff attached to the leg over the left calf muscle, and end-tidal carbon dioxide attached to nasal prongs.

**Figure 2:**
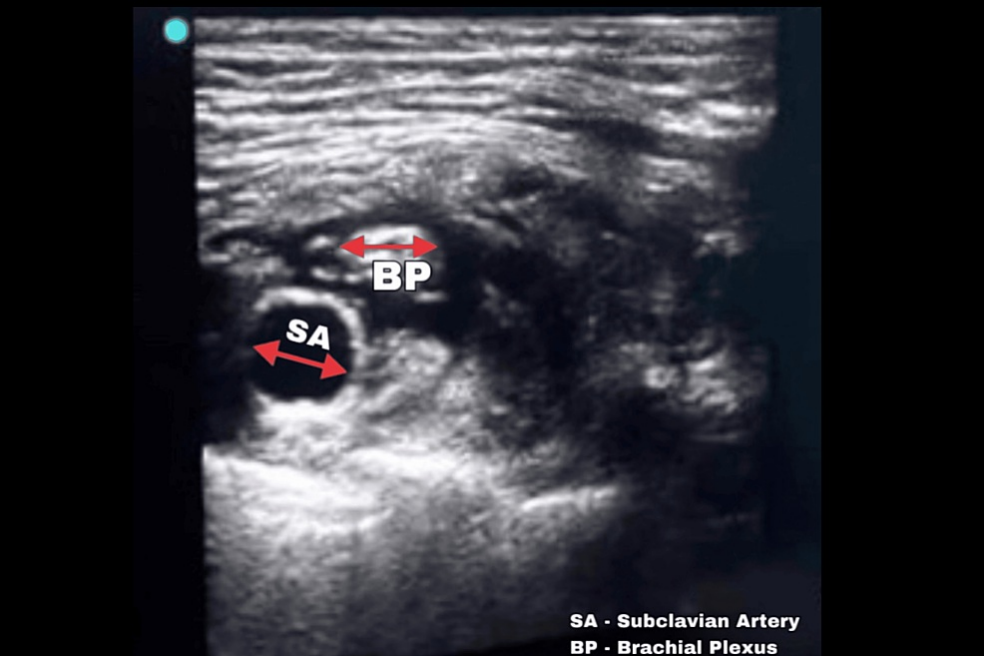
The ultrasound image depicting the post-supraclavicular local anesthetic spread around the brachial plexus.

We utilized a linear array 13-6 MHz transducer of Sonosite Edge II (Bothell, WA: FUJIFILM Sonosite, Inc.) for ultrasound-guided supraclavicular brachial plexus block. Following aseptic skin preparation and local anesthesia using 2% lignocaine, we positioned the probe in the supraclavicular fossa and identified the subclavian artery. The brachial plexus was located as hypoechoic nodules, superiorly and laterally to the subclavian artery, and enclosed by a hyperreflective fascial sheath. The puncture was performed in-plane (IP) along the long axis of the probe, moving from lateral to medial using echogenic single shot nerve block Stimuplex (Tokyo, Japan: B. Braun SE). Finally, we injected 20 mL of 0.5% plain bupivacaine around the brachial plexus bundle after confirming a negative aspiration test (Figure 2). The block adequacy was tested by motor and sensory block. Response to pinprick was used to assess sensory block. The areas supplied by median, radial, ulnar, musculocutaneous, and medial cutaneous nerves of the forearm were assessed individually and noted. In the unfortunate event of sparing respiratory compromise or inadequate nerve block, general anesthesia was planned to be administered. It took 12 minutes for a complete sensory blockade and 18 minutes for a complete motor blockade. The anesthesia injection to surgical incision time was 20 minutes.

The patient underwent a K-wire fixation of the wrist deformity and debridement. The arterial tourniquet was not used as it was a planned closed procedure. Intra-operative monitoring indicated stable hemodynamics without any respiratory compromise throughout the procedure. The patient reported no pain or discomfort, no rescue analgesic was used, and the surgery was successfully completed. The patient was shifted to the post-anesthesia care unit for observation for 2 hours, following which she was shifted to the ward. The patient was followed up for analgesic supplementation, motor and sensory effects of the nerve block in the post-operative period, hemodynamic parameters, and respiratory status for five days and subsequently discharged.

## Discussion

Transverse myelitis is characterized by localized inflammation affecting one or multiple spinal cord levels. Its symptoms encompass motor, sensory, and autonomic dysfunction. Motor problems often manifest as the rapid development of paraparesis, which may initially involve the upper extremities with flaccidity, followed by spasticity [7].

The supraclavicular approach to brachial plexus block is a regional anesthetic technique used as an alternative or adjunct to general anesthesia for upper extremity surgeries. Its anatomical advantage lies in blocking the brachial plexus at a site where its components are closely clustered, enabling a single injection point and a rapid onset of effect [8]. In comparison with the blind technique using anatomical landmarks, the ultrasound-guided technique of nerve block has minimal complications and increased success rates due to easy image acquisition relating to the superficial location of the brachial plexus and identifying the pleura thus minimizing complications [9,10].

As the lateral position was more convenient for the transverse myelitis patient, a supraclavicular brachial plexus block was attempted in our patient's agreeable lateral decubitus position with an in-plane lateral to medial approach. Additionally, Chen et al. observed that ultrasound-guided brachial plexus block in lateral decubitus position with in-plane lateral approach has minimal complications like pneumothorax compared to other positions [11]. In this study, they have compared the supraclavicular approach to the brachial plexus block in the supine, body turned 45° sideways and in the lateral decubitus position. This was because the distance from the pleura to the inferior trunk increased significantly and there was also a positive correlation with BMI.

Careful selection of the correct anesthetic technique and personalized positions are important in such circumstances. In this case, the supraclavicular brachial plexus block performed in a lateral position offered a safe and effective targeted anesthesia minimizing the risks of general anesthesia in this patient.

## Conclusions

This study aimed to shed light on the significance of atypical and personalized positioning for a regional anesthesia technique in patients with neurological conditions. Supraclavicular brachial plexus block in lateral decubitus position proved a viable option in a patient with transverse myelitis and rheumatoid arthritis undergoing left wrist deformity.

## References

[1] (2002). Proposed diagnostic criteria and nosology of acute transverse myelitis. Neurology.

[2] Awad A, Stüve O (2011). Idiopathic transverse myelitis and neuromyelitis optica: clinical profiles, pathophysiology and therapeutic choices. Curr Neuropharmacol.

[3] Hambly PR, Martin B (1998). Anaesthesia for chronic spinal cord lesions. Anaesthesia.

[4] Hebl JR, Horlocker TT, Schroeder DR (2006). Neuraxial anesthesia and analgesia in patients with preexisting central nervous system disorders. Anesth Analg.

[5] Brown DL, Cahill DR, Bridenbaugh LD (1993). Supraclavicular nerve block: anatomic analysis of a method to prevent pneumothorax. Anesth Analg.

[6] Neal JM, Gerancher JC, Hebl JR, Ilfeld BM, McCartney CJ, Franco CD, Hogan QH (2009). Upper extremity regional anesthesia: essentials of our current understanding, 2008. Reg Anesth Pain Med.

[7] Simone CG, Emmady PD (2023). Transverse myelitis. StatPearls [Internet].

[8] Franco CD, Vieira ZE (2000). 1,001 subclavian perivascular brachial plexus blocks: success with a nerve stimulator. Reg Anesth Pain Med.

[9] Hanumanthaiah D, Vaidiyanathan S, Garstka M, Szucs S, Iohom G (2013). Ultrasound guided supraclavicular block. Med Ultrason.

[10] Kurdi MS, Agrawal P, Thakkar P, Arora D, Barde SM, Eswaran K (2023). Recent advancements in regional anaesthesia. Indian J Anaesth.

[11] Chen CP, Hsu CC, Cheng CH, Huang SC, Chen JL, Lin SY (2021). The lateral decubitus body position might improve the safety of ultrasound-guided supraclavicular brachial plexus nerve block. J Pain Res.

